# The Hidden Burden: Gastrointestinal Involvement in Lysosomal Storage Disorders

**DOI:** 10.3390/metabo15060361

**Published:** 2025-05-29

**Authors:** Vincenza Gragnaniello, Chiara Cazzorla, Daniela Gueraldi, Andrea Puma, Christian Loro, Alberto B. Burlina

**Affiliations:** Division of Inherited Metabolic Diseases, Department of Women’s and Children’s Health, University Hospital of Padova, 35128 Padova, Italy; chiara.cazzorla@aopd.veneto.it (C.C.); daniela.gueraldi@aopd.veneto.it (D.G.); andrea.puma@aopd.veneto.it (A.P.); christian.loro@aopd.veneto.it (C.L.); alberto.burlina@unipd.it (A.B.B.)

**Keywords:** lysosomal storage diseases, Fabry disease, Gaucher disease, Pompe disease, Niemann–Pick type C, lysosomal acid lipase deficiency, mucopolysaccharidoses, gastrointestinal involvement, bowel disease, enteropathy

## Abstract

Background: Lysosomal storage disorders (LSDs) are rare inherited metabolic diseases characterized by defects in lysosomal enzyme function or membrane transport. These defects lead to substrate accumulation and multisystemic manifestations. This review focuses on gastrointestinal (GI) involvement in LSDs, which is a significant but often overlooked aspect of these disorders. Methods: A comprehensive literature review was conducted to examine the pathophysiology, clinical presentation, diagnosis and management of GI manifestations in several LSDs, including Fabry disease, Gaucher disease, Pompe disease, Niemann–Pick disease type C, mucopolysaccharidoses and Wolman disease. Results: The pathogenesis of GI involvement in LSDs varies and encompasses substrate accumulation in enterocytes, mesenteric lymphadenopathy, mass effects, smooth muscle dysfunction, vasculopathy, neuropathy, inflammation and alterations to the microbiota. Clinical presentations range from non-specific symptoms, such as abdominal pain, diarrhea and malabsorption, to more severe complications, such as protein-losing enteropathy and inflammatory bowel disease. Diagnosis often requires a high level of suspicion, as GI symptoms may precede the diagnosis of the underlying LSD or be misattributed to more common conditions. Management strategies include disease-specific treatments, such as enzyme replacement therapy or substrate reduction therapy, as well as supportive care and targeted interventions for specific GI complications. Conclusions: This review highlights the importance of recognizing and properly managing GI manifestations in LSDs to improve patient outcomes and quality of life. It also emphasizes the need for further research to develop more effective treatments for life-threatening GI complications associated with these rare genetic disorders.

## 1. Introduction

Lysosomal storage disorders (LSDs) are rare inherited metabolic diseases characterized by defects in the function of specific enzymes within cellular organelles called lysosomes. These enzymes are essential for processing macromolecules, including degrading proteins (e.g., hydrolases), transporting membranes and producing chaperone proteins. When these enzymes are deficient, undigested substrates accumulate within lysosomes, leading to cellular dysfunction, tissue damage and various clinical manifestations.

The clinical features of LSDs vary depending on the degree and type of enzyme deficiency, the type and extent of substrate accumulation and the affected tissues. Manifestations range from mild to severe multiorgan dysfunction, which can result in significant limitations for patients and their families [1].

Gastrointestinal (GI) involvement in lysosomal diseases can occur directly, due to substrate accumulation, or indirectly, due to the involvement of other systems, such as neuropathy and vasculopathy, or as a result of certain medications. The response to available treatments (enzyme replacement therapy, substrate inhibitors and chaperones) varies.

In some cases, GI symptoms can be frequent and appear early, as in Fabry disease and Wolman disease. Recognizing these symptoms can help to avoid diagnostic delays. In other conditions, such as Niemann–Pick type C and Gaucher disease, GI symptoms appear later in the disease course and indicate severe disease with an unfavorable prognosis. Understanding the pathogenic mechanisms in these cases can be useful for identifying appropriate treatment strategies.

GI symptoms are often underdiagnosed, underreported or misdiagnosed as concomitant conditions due to their non-specific nature and lack of awareness among healthcare professionals. The aim of our review is to identify the main GI manifestations of LSDs, their pathogenesis and their treatment, in order to raise awareness of the more common forms and provide an overview of how to manage these rare manifestations. Specifically, we analyzed GI involvement in Fabry disease, Gaucher disease, Pompe disease, Niemann–Pick type C, mucopolysaccharidosis and Wolman disease. Initially, acid sphingomyelinase deficiency (ASMD), mucolipidosis, α-mannosidosis and fucosidosis were included in the search, but were later excluded due to a lack of evidence. GI manifestations affecting the liver or mouth (e.g., hepatomegaly or abnormal dentition) were excluded.

## 2. Relevant Sections

### 2.1. Fabry Disease

Fabry disease (FD, OMIM #301500) is an X-linked storage disorder caused by a deficiency of the enzyme α-galactosidase A. This leads to the accumulation of glycolipids, such as globotriaosylceramide (Gb3) and globotriaosylsphingosine (lysoGb3), in various cell types, including vascular, cardiac, renal, nerve and skin cells [2,3]. FD is one of the most prevalent lysosomal storage disorders, with an incidence of 1 in 4000 male newborns according to newborn screening [4,5,6].

The classic form of FD manifests in childhood with symptoms including neuropathic pain, hypohidrosis, angiokeratomas, cornea verticillata and GI issues. In adulthood, patients may experience renal failure, cardiac complications and neurological impairment. A late-onset (LO) form occurs in the fourth to seventh decades of life, with patients primarily showing a cardiac, renal and neurological involvement. Due to the random X-inactivation, females can exhibit varying degrees of severity [3].

A diagnosis can be made through measuring the enzyme activity in males, identifying glycosphingolipid accumulation (Gb3 and, in particular, its deacylated form, lysoGb3) and conducting a genetic analysis [5].

While central nervous system, heart and kidney diseases can be life-threatening, GI symptoms can be significantly disabling [7,8]. The Fabry Outcome Survey (FOS), based on data from 1453 patients, reported a 51% prevalence of GI symptoms [9]. GI symptoms may be the first clinical manifestation of FD, even in children [10,11,12], and correctly recognizing them can prevent a delay in diagnosis [13].

The most common GI symptom in FD is abdominal pain, affecting up to one-third of patients. Patients report swelling, cramps, burning pain and abdominal discomfort. The pain often worsens after meals and during periods of stress [14]. Diarrhea is the second most common GI symptom, affecting 20% of patients. A subgroup of patients, mainly women, suffer from constipation. Less common, yet still significant, symptoms include vomiting, nausea and early satiety, which can lead to serious dietary restrictions [15]. However, the mean BMI is similar in patients with and without GI symptoms [16]. These symptoms significantly impact quality of life [17,18].

It should be noted that GI symptoms were not represented in the FD severity scores (MSSI) [19]. A FABry disease-specific Patient-Reported Outcome-Gastrointestinal (PRO-GI) questionnaire should be used to assess the severity of GI symptoms and the effects of therapies [20].

Because GI signs and symptoms are non-specific, this can contribute to long diagnostic delays and misdiagnosis. In patients with a long-term history of unexplained GI symptoms, FD should be considered as a potential diagnosis [13,15]. Pathological findings are specific. If no other cause of the observed symptoms is identified during endoscopy (gastroscopy or colonoscopy), biopsies with appropriate anti-Gb3 immunostaining [21] or electron microscopy [22] can reveal Gb3 deposits in the epithelial cells of the gut in patients with classical FD, thus supporting the diagnosis [23].

**Figure 1:**
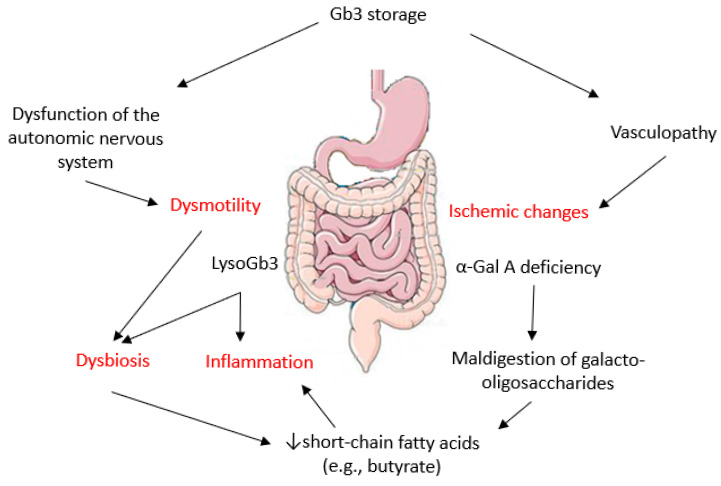
Pathogenic mechanisms of gastrointestinal involvement in Fabry disease.

Several mechanisms contribute to the pathogenesis of GI symptoms in FD (Figure 1). Gb3 accumulation in enteric neurons and endothelial cells can cause autonomic nervous system dysregulation, adversely affecting gut motility [14,18,24] and GI vasculopathy [25,26], respectively. LysoGb3 triggers tissue inflammation and modifies the bacterial composition of the human colon microbiota [27]. Dysbiosis alters the formation of short-chain fatty acids (SCFAs), including butyrate, which has anti-inflammatory properties and reduces systemic vascular injury [28]. Furthermore, dysbiosis can impair the supply of energy to the colonic epithelium and increase its permeability, resulting in a ‘leaky gut’ [29,30]. Beyond local effects, dysbiosis disrupts the delicate microbiota–gut–brain axis [31], highlighting its systemic impact. Finally, a lack of α-galactosidase A may result in the malabsorption of nutrients (e.g., oligosaccharides) within the gut [23,32].

According to European recommendations, the presence of GI symptoms that are not successfully treated with symptomatic therapy indicates the need for disease-specific therapy [33]. A study by Frustaci et al. found that two years of ERT completely clears enterocytes of Gb3 inclusions [34]. However, this therapy is not always effective: more than half of patients treated with ERT continue to experience GI symptoms, and some patients even develop new GI symptoms during treatment [14].

Data on migalastat are limited; however, it resulted in a significant reduction in GI symptoms after 24 months of treatment in both treatment-naïve patients and those previously treated with ERT [35,36].

Dietary interventions and supplements may offer additional benefits in managing GI symptoms. FODMAPs (fermentable oligosaccharides, disaccharides, monosaccharides and polyols) are small carbohydrates found in many common foods [22]. They are poorly absorbed in the small intestine and can pass into the colon, where they increase the water in the lumen through osmotic activity and induce gas production due to bacterial fermentation. This can lead to luminal distension, GI symptoms and dysbiosis [37]. As a low-FODMAP diet is already used to treat irritable bowel syndrome (IBS), patients with FD may benefit particularly from it, since many FODMAPs require the enzyme α-galactosidase A for correct digestion [23]. A study evaluating the effectiveness of a low-FODMAP diet for patients with FD and GI symptoms demonstrated reduced indigestion, diarrhea and constipation in seven adults. However, adherence can be challenging, and long-term results have not yet been evaluated [38].

Dietary supplements containing orally delivered α-galactosidase A seem beneficial. A small study of seven patients reported improvements in abdominal pain and diarrhea after taking a daily dose of 1800 U of α-galactosidase A for 90–180 days, with no adverse effects reported. This approach supports the proper digestion of galacto-oligosaccharides and may deplete intestinal lyso-Gb3, which could result in a reduced proinflammatory status and dysbiosis. If absorbed, it may also lead to a Gb3 depletion in endothelial cells and neurons [39]. Further research is needed to evaluate the long-term efficacy of these approaches.

While numerous studies have investigated GI symptoms in FD and their pathogenesis, the main limitation is the small number of studies that have examined the impact of available therapies, both specific and symptomatic.

Table 1 shows the main interventional clinical trials for FD that include the evaluation of GI symptoms among their endpoints [Clinicaltrials.gov, last accessed on 20 May 2025].

### 2.2. Gaucher Disease

Gaucher disease (GD) is caused by a deficiency of acid β-glucosidase, which leads to the accumulation of glucosylceramide (Gb1) and glucosylsphingosine (lysoGb1) in macrophages (Gaucher cells) within the reticuloendothelial system [40]. There are three clinical phenotypes of GD. Type 1 (non-neuronopathic) GD (GD1) accounts for over 90% of reported GD cases (OMIM #230800) and is characterized by common manifestations including anemia, thrombocytopenia, splenomegaly, hepatomegaly and bone lesions. Type 2 GD (GD2) (OMIM #230900) is characterized by acute and severe neurological impairments, as well as visceral symptoms. Type 3 GD (GD3) (OMIM #231000) is associated with a chronic neurological impairment of a variable but lesser severity [41,42,43].

A diagnosis can be made through measuring enzyme activity, identifying glycosphingolipid accumulation (lysoGb1) and performing a genetic analysis [44].

GI manifestations related to GD are rare. Two main mechanisms have been described for GI involvement in GD: abdominal Gaucheroma and mucosal infiltration.

Gaucheroma comprises Gaucher cells and typically manifests in lymph nodes within the mesenteric and mediastinal regions [45]. The clinical manifestations of mesenteric Gaucheroma can vary greatly, ranging from being asymptomatic in mild cases to presenting as protein-losing enteropathy (PLE) in severe cases [46,47].

**Figure 2:**
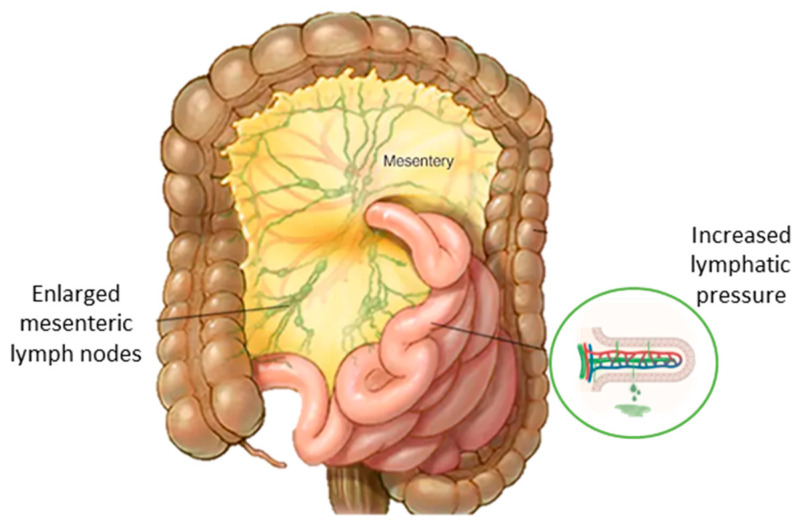
Pathogenic mechanism of protein-losing enteropathy in GD. Enlarged mesenteric lymph nodes block mesenteric lymphatic outflow, resulting in increased lymphatic pressure and intestinal lymphangiectasia.

PLE occurs when enlarged mesenteric lymph nodes block the mesenteric lymphatic outflow, resulting in increased lymphatic pressure and intestinal lymphangiectasia (Figure 2) [48,49,50,51]. Symptoms include abdominal pain, distension, diarrhea, hypoproteinemia, oedema, weight loss and malnutrition. Complications may include calcification, thrombosis, ascites and electrolyte disturbances [52].

Mesenteric Gaucheroma can initially be suspected through ultrasound [53]. However, CT and MRI are the gold standards for confirming and measuring enlarged nodes. CT can reveal calcified lymph nodes [53,54,55]. Although a biopsy should not be performed routinely, it may be useful for confirming the pathology if a progressive enlargement is observed or malignancy is suspected [53]. Lymph node biopsies typically reveal Gaucher cells, which are characterized by histiocytosis and the expression of CD163, CD68 and VEGF [56]. There are rare reports of calcification and fibrosis [57,58]. A diagnosis of PLE can be confirmed by increased fecal concentrations of alpha-1-antitrypsin [59].

Only a few cases of Gaucheroma with PLE have been reported [48,49,50,53,54,55]. It primarily occurs in neuropathic phenotypes, especially those carrying the p.Leu483Pro variant in the *GBA1* gene. Additional contributing factors include the development of antibodies against the enzyme, which can reduce treatment efficacy, and splenectomy, which can lead to the deposition of substrates in other reticuloendothelial organs. PLE often signals disease advancement and predicts a challenging prognosis [54].

There is currently no effective treatment for Gaucher tumors with PLE. ERT has a limited efficacy in these cases, suggesting that these manifestations are caused by substrate accumulations in sequestered sites that are difficult for ERT to penetrate [48,53]. Emerging evidence suggests that small-molecule SRTs may be able to penetrate sequestered sites more readily than ERT. However, results are conflicting [48,51,54,55], and more research is required.

Supportive treatments include intermittent albumin infusion, fluid and electrolyte replacement, a high-protein diet supplemented with medium-chain lipids and parenteral nutrition [48]. Budesonide has been used in cases of PLE [51]. Surgical intervention, such as serial partial lymph node excisions with adhesiolysis, has been reported, but is generally not indicated due to the high risk of complications [50].

GI mucosal infiltration is a rare complication of GD. The GI tract mucosa may be resistant to ERT due to the relative scarcity of mannose receptors [60]. Kim et al. reported on identical twin siblings with GD1 who were on ERT and who developed epigastric discomfort and weight loss. An upper GI endoscopy revealed multiple yellowish nodular lesions on the duodenum, consisting of Gaucher cell infiltration. One sibling treated with eliglustat, which can penetrate the GI mucosa more effectively than ERT, showed improvement [61].

Other cases of mucosal involvement have been reported, including the diffuse mucosal thickening of small bowel loops in a 24-year-old patient with GD3 [62] and a small bowel mucosal involvement proven by biopsies in children [63]. A case of periappendicitis attributed to the overwhelming infiltration of adjacent lymph nodes, periappendiceal tissue and the outer layers of the appendiceal wall by Gaucher cells has also been reported [64].

Finally, a few cases of GI hemorrhage associated with gastric ulcers or ileal lymphoid hyperplasia have been reported. These include fatal GI bleeding in an 11-month-old with GD2 due to a gastric ulcer infiltrated by Gaucher cells [17], bleeding from the terminal ileum associated with lymphoid hyperplasia in an adult with GD [65] and colonic bleeding from a juvenile polyp infiltrated with Gaucher cells in a 10-year-old girl with GD3 [66].

Although GD is one of the most common lysosomal storage disorders, GI complications are rare and tend to occur in patients with more severe forms of the disease. Therefore, the literature is limited to individual case reports or small case series, frequently concerning patients with neuropathic forms.

### 2.3. Pompe Disease

Pompe disease (PD), also known as glycogen storage disease type II (OMIM #232300), is caused by a deficiency of the enzyme acid alpha-glucosidase (GAA). This leads to glycogen accumulating throughout the body, particularly in the heart and skeletal muscles [67]. The disease is categorized into two main forms: infantile-onset Pompe disease (IOPD) and late-onset Pompe disease (LOPD). Patients with IOPD present with cardiomyopathy and hypotonia in the first months of life, and if left untreated, they die from cardiorespiratory complications before reaching the age of two [68,69]. Patients with LOPD, on the other hand, present with slowly progressive myopathy and respiratory failure, with a variable symptom onset [70,71].

A diagnosis can be made through measuring enzyme activity and performing a genetic analysis [71].

There is growing evidence of a smooth muscle involvement in individuals with PD, including in the GI tract. The accumulation of glycogen, accompanied by vacuolisation, autophagy and fibrosis, in the smooth muscles of the lamina muscularis throughout the GI tract has frequently been demonstrated in mouse models of PD, as well as in both infantile and adult-onset human cases [72,73,74,75,76,77]. This glycogen accumulation explains the intestinal dysmotility and impaired bowel control observed in patients with both IOPD and LOPD [78].

GI difficulties are common in IOPD. A retrospective chart review of 168 IOPD patients found that over 50% presented with feeding difficulties or a failure to thrive at a median age of four months [70]. Clinically significant dysphagia is a common finding in IOPD survivors [79,80,81].

Feeding problems in infants with PD may be related to swallowing difficulties, as well as gastro-esophageal reflux disease (GERD). Deficits contributing to impaired swallowing include decreased sucking strength, oral dysmotility and the delayed initiation of the swallowing reflex. In the pharyngeal phase, the residue of liquids after swallowing increases the risk of aspiration [82]. GERD occurs when the lower esophageal sphincter is incompetent, which increases the risk of aspiration [83]. It should be noted that Nissen fundoplication should be carefully considered due to its limited success in patients with underlying myopathy, its lengthy procedure and the high anesthesia risk in infants with PD [82].

In LOPD, GI manifestations include abdominal pain, feeding and swallowing difficulties, gastroesophageal reflux, postprandial bloating, early satiety, abdominal discomfort, chronic diarrhea, constipation and poor weight gain [84,85,86,87,88,89].

A significant proportion of LOPD patients (76% in one study) report at least one GI problem, with 40% considering their GI symptoms to be among the top three factors affecting their quality of life. Interestingly, GI manifestations often precede the LOPD diagnosis (42%), with the age of onset ranging from childhood to the 40 s [90].

One interesting GI manifestation observed in LOPD patients is Chilaiditi’s sign, defined as the hepatodiaphragmatic interposition of the intestine. This rare anatomical abnormality can cause recurrent abdominal pain or even colonic volvulus. LOPD patients may be more prone to developing Chilaiditi’s sign due to diaphragmatic factors such as atrophy and fat infiltration, as well as bowel factors such as an abnormally dilated bowel [91].

Patients with both IOPD and LOPD experience significantly more urgency and incontinence compared to age- and gender-matched controls, with a complex pathophysiology involving the striated and smooth muscles of the pelvic floor, as well as the potential involvement of the lower motor neurons or the autonomic nervous system. Diagnostic tests have revealed myogenic patterns in the anal sphincter and a reduced pressure in both the external (striated muscle) and internal (smooth muscle) anal sphincters [92].

**Figure 3:**
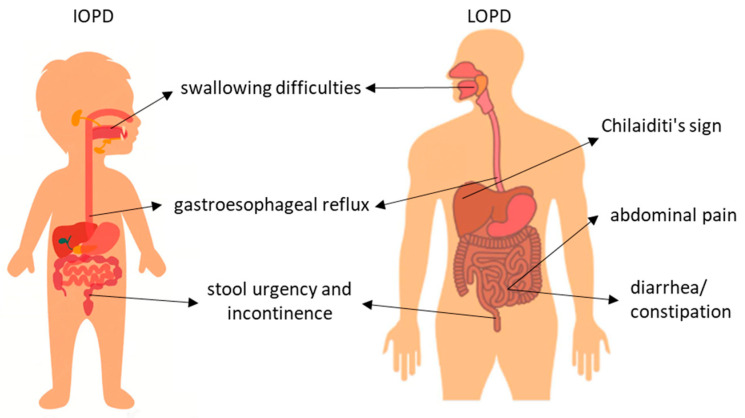
More frequent gastrointestinal symptoms reported in patients with IOPD and LOPD.

Figure 3 shows the most common GI symptoms reported in patients with IOPD and LOPD. 

Case series have reported substantial improvements in GI symptoms following the initiation of ERT [84,86,88,89].

However, despite being on ERT, some LOPD patients report no changes in their GI symptoms over time. This discrepancy suggests that the ERT may be insufficient to effectively target GI symptoms, possibly due to a reduced uptake in the scarred or fibrotic muscle tissue in the late phase of PD [90].

These findings highlight the importance of a comprehensive GI assessment and management in both LOPD and IOPD patients. They also highlight the need for further research into effective treatments for these symptoms, which significantly impact patients’ quality of life.

It should be considered that the existing literature refers only to alglucosidase therapy. However, two other enzyme therapies have recently been developed: avalglucosidase and cipaglucosidase alpha. Therefore, the impact of these new therapies, as well as newborn screening which is becoming increasingly widespread worldwide, will need to be evaluated in the future.

### 2.4. Niemann–Pick Type C

Niemann–Pick type C (NPC) (OMIM #257220) is a neurodegenerative disorder that is caused by variants in either the NPC1 gene (in 95% of cases) or the NPC2 gene. These variants disrupt the trafficking of lipids in lysosomes and endosomes, resulting in the accumulation of unesterified cholesterol and sphingolipids [93]. The clinical presentation of NPC is variable. In neonatal-onset NPC, hepatosplenomegaly, jaundice and liver or respiratory failure are often the initial symptoms [94]. However, the most common presentation is neurological, including seizures, speech impairment, early-onset dementia, ataxia, dysphagia and vertical supranuclear gaze palsy in early to late childhood, as well as dystonia and frank schizophrenia in adulthood [95,96]. The diagnosis is based on the plasma biomarker lysoSM-509 and a genetic confirmation [97].

Approximately 7% of NPC patients reportedly develop inflammatory bowel disease (IBD), particularly Crohn’s disease (CD) [98,99]. A cohort study of 14 patients with NPC1 defects who presented with IBD found that the mean age at IBD diagnosis was 12.8 years, which is significantly earlier than in typical IBD cases. NPC patients show an increased susceptibility to fistulizing colitis with granuloma formation and perianal disease and are difficult to treat [100,101,102].

The pathophysiology of NPC-associated IBD involves two main factors. Firstly, the NPC1 deficiency inhibits the STING (stimulator of interferon genes) transport and degradation in lysosomes, thereby enhancing STING signaling and promoting intestinal inflammation through inflammatory cytokine production [30,103]. Secondly, NPC1 mutations induce a defect in autophagy, leading to impaired NOD2-mediated bacterial killing by macrophages [98]. The persistence of bacteria in the gut wall leads to an increased cytokine response and potential granuloma formation [98,104]. The synergistic effect of NPC1 and NOD2 mutations has also been observed in a patient with hepatomegaly, CD and neurological manifestations, who carries a single variant in both genes. This suggests that both genes can independently lead to immune system activation [105].

**Figure 4:**
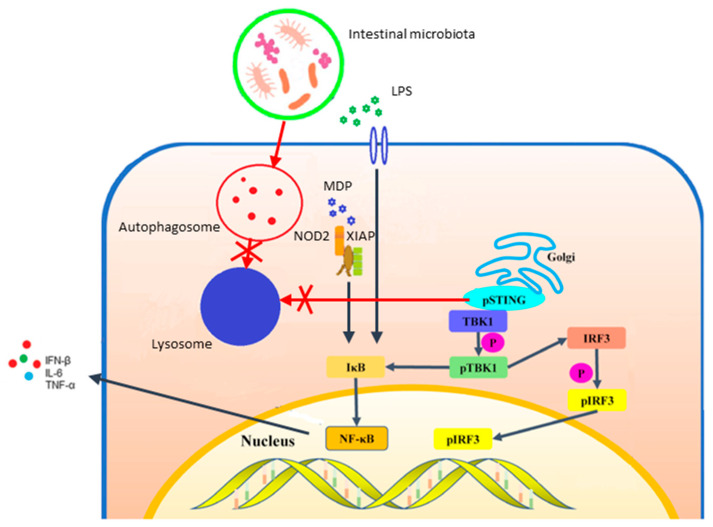
Pathogenic mechanisms of NPC-associated inflammatory bowel disease. The pathophysiology of NPC-associated IBD involves enhanced STING signaling and impaired NOD2-mediated killing of bacteria. LPS: lipopolysaccharide and MDP: muramyl dipeptide.

Figure 4 summarizes the pathophysiology of NPC-associated IBD.

Treatment implications include inducing autophagy to improve bacterial handling [98] and using biologic therapy to target cytokines [106]. A case report of a 13-year-old female treated with a dual biological therapy, including infliximab (anti-TNFα) and ustekinumab (anti-IL-12/IL-23), showed promising results [107].

Given the incidence of IBD in patients with NPC, measuring fecal calprotectin as an early indicator of GI involvement could be considered during follow-up.

In addition to IBD, patients with NPC may develop a disaccharidase deficiency, contributing to symptoms such as diarrhea, nausea, bloating, abdominal pain and weight loss [108,109]. This is due to the disruption of cholesterol- and sphingolipid-enriched lipid rafts, which are essential for the proper sorting of GI enzymes, such as sucrase–isomaltase and dipeptidyl peptidase, to the apical surface of the intestinal epithelium [108,110,111]. A case study of a 15-year-old girl with NPC confirmed reduced activities of these disaccharidases in a duodenal biopsy [112].

It is also important to note that miglustat, which is used in the treatment of NPC as an SRT, can bind directly to the catalytic sites of intestinal α-glucosidases and inhibit their activity, potentially exacerbating GI symptoms [113].

Considering the high incidence and early onset of IBD in patients with NPC, the current limitations of the literature are the lack of early markers of intestinal involvement and effective therapies.

### 2.5. Mucopolysaccharidoses

Mucopolysaccharidoses (MPS) are a group of diseases caused by a deficiency in the activity of specific lysosomal enzymes involved in the degradation of mucopolysaccharides (also known as glycosaminoglycans or GAGs). Symptoms can include facial dysmorphology, frequent upper respiratory tract infections, hepatomegaly, splenomegaly, joint stiffness and bone deformity, cardiac disease and neurological involvement [114,115,116]. Diagnosis is based on a quantitative and qualitative assay of urinary GAGs, an analysis of enzyme deficiency (specific to each form) and a genetic analysis [116].

GI involvement is probably underestimated. Thomas et al. reviewed the causes of death in MPS III patients recorded on 221 UK death certificates (1957–2020) and found that 5.9% of cases listed GI conditions. Diarrhea and dysphagia were the most common conditions reported in the literature, followed by constipation and a loss of bowel control/fecal incontinence. Besides deaths related to feeding problems, other GI conditions that contribute to MPS III mortality include GI failure, GI bleeding, gastroenteritis and paralytic ileus [117]. A chronic intestinal pseudo-obstruction (CIPO) has been reported in two patients with MPS I [118,119].

It has been speculated that GAGs may infiltrate the human GI tract. The evaluation of the GI tract in MPS IIIa mice has demonstrated lysosomal GAG accumulation in the lamina propria of the villi of the duodenum, jejunum and ileum, as well as increased lysosomal storage throughout the GI system [120]. Autopsies on humans have revealed GAG accumulation and vacuolization in the pyloric ring and the extrinsic nerves of the Auerbach nerve plexuses, which may explain abnormalities in GI peristalsis [121,122].

The gut microbiota appears to play an important role in MPS. Bacterial flora metabolize GAGs [123,124,125,126]. Accumulated GAGs could provide a favorable medium for microbial growth and might impair the production and/or action of IgA, thereby making patients more susceptible to microbial infections [122]. Wegrzyn et al. demonstrated that atypical microbial infections of the digestive tract may contribute to diarrhea in MPS I and do not recur after the initiation of ERT, indicating that diarrhea results directly or indirectly from GAG accumulation [122].

Herranz et al. studied two siblings with MPS IIIa who had the same gene variants and diet. They found that the patient with GI symptoms had a slightly lower alpha diversity and lower Bacteroides abundance. This patient had the highest fecal levels of HS, despite having lower urinary levels and a higher enzyme activity. They also had lower levels of short-chain fatty acids (SCFAs) [127]. SCFAs, produced by the gut microbiota from fiber digestion, regulate inflammation and mitochondrial function, thereby reducing oxidative stress [128,129,130].

Finally, localized GAG storage may mechanically obstruct intestinal lymphatics. Ferrandez et al. and Sibilio et al. reported two patients with MPS IIIb who had chronic diarrhea and endoscopic and histological findings that were consistent with intestinal lymphangiectasia. A low-fat diet supplemented with medium-chain triglycerides (MCTs) led to the persistent improvement of the diarrhea [131,132].

It should be noted that most articles on GI involvement concern MPS III, which primarily affects the neurological system and for which systemic enzyme replacement therapy is unavailable.

### 2.6. Wolman Disease

Wolman disease (WD) is characterized by the complete loss of function of lysosomal acid lipase, which is responsible for the hydrolysis of triglycerides and cholesterol esters [133,134]. WD typically presents in infancy and is characterized by vomiting, diarrhea, hepatosplenomegaly with cirrhotic evolution, calcific deposits in the adrenal glands, significant abdominal distension, anemia and failure to thrive due to malabsorption. Psychomotor development is also delayed. Without treatment, the disease is almost always fatal before the age of one, with malabsorption and diarrhea being the main causes of death [133,134,135,136]. A diagnosis can be made through measuring enzyme activity and performing a genetic analysis [137]. ERT (sebelipase alpha) is available [138].

In WD, malabsorption, bowel dilatation, bowel-wall thickening and ulceration are due to the infiltration of the lamina propria by foamy histiocytes [139]. Abdominal ultrasounds can reveal the oedema of the small bowel mucosa. The infiltrated lamina propria becomes hyperechoic and indistinguishable from the adjacent submucosa. The subsequent abnormal peristalsis may result in acute or transient intussusception [140,141].

Diffuse pancolic mural thickening can be observed on abdominal CT [142] and has been described as early as the first month of life [143].

Endoscopically, the picture is characterized by yellowish, elevated and coalescent nodular lesions in the duodenum. The light microscopy of duodenal biopsies reveals histiocytes with enlarged cytoplasm in the lamina propria and submucosa that infiltrate the villi [144]. This has sometimes enabled a diagnosis to be made, as in the case of a 29-year-old woman with intermittent diarrhea and abdominal pain for seven years [138], and it has also been reported in a pediatric patient aged eight years and ten months [144]. In a two-month-old patient, a GI endoscopy revealed pale, bare jejunal mucosa with a reduced number of mucosal folds [135]. Similar findings have been reported in the colonic mucosa [142].

A case of Crohn’s disease presentation has been reported. A 39-month-old girl presented with multiple erythematous and macerated fissures in the perianal area. The GI endoscopy revealed multiple duodenal and colonic aphthous ulcers. A histological examination showed the expansion of the lamina propria with foamy histiocytes and xanthoma cells, consistent with WD [145].

The main limitation is that, as it is an ultra-rare disease, the reported cases are anecdotal.

Table 2 summarizes the main symptoms, pathogenesis, diagnostic approach and management of the gastrointestinal involvement in the lysosomal diseases analyzed in this review.

## 3. Conclusions and Future Directions

LSDs are multisystemic disorders that can affect the GI tract. The pathogenesis varies depending on the specific disease and can be related to the accumulation of substances in enterocytes, mesenteric lymphadenopathy, the mass effect, the involvement of parietal smooth muscle, vasculopathy, neuropathy, inflammation and alterations to the microbiota.

Symptoms are rarely specific and can resemble those of more common diseases. Although the specific treatment for LSDs is effective, it does not always allow for a complete resolution of symptoms, so supportive measures may be necessary.

Maintaining a high degree of awareness is essential to avoid delaying the initial diagnosis by looking for systemic symptoms and to avoid delaying the diagnosis of complications, which may benefit from a more complex therapeutic approach.

Future directions:

- Identify specific biomarkers for the early detection of GI involvement in LSDs.
- Enhance ERT targeting and develop alternative therapies for life-threatening symptoms that are currently difficult to manage (e.g., intestinal lymphangiectasia in GD).
- Evaluate the efficacy of anti-inflammatory and antioxidant therapies in LSDs with an inflammatory pathogenesis (e.g., Crohn’s-like in NPC) or where inflammation exacerbates symptoms (e.g., in Fabry and Gaucher diseases).
- Assess targeted microbiome interventions in FD, NPC and MPS.

## Figures and Tables

**Table 1:** Overview of clinical trials with gastrointestinal endpoints in Fabry disease.

ClinicalTrials.gov ID	Drug	Route of Administration	Phase	Type of Therapy	Eligible Ages and Sexes	GI Endpoint	Study Completion
NCT06819514	EXG110	IV	Phase 1/2	Gene therapy	Adult, M/F	Gastrointestinal Symptom Rating Scale	2028-03
NCT06114329	AL01211	Orally	Phase 2	SRT	Adult, M, classic form	Gastrointestinal symptom diary	2026-06
NCT05710692	PRX-102 (pegunigalsidase alfa)	IV	Phase 2/3	ERT	Child (>13 yrs)/Adult, M/F	Gastrointestinal Symptom Rating Scale (GSRS) scores (adult), Gastrointestinal Symptoms (PedsQL-GI) Questionnaire scores (child)	2028-03

**Table 2:** The symptoms, pathogenesis, diagnostic approach and management of the gastrointestinal involvement in the lysosomal diseases.

GI Manifestations/When to Suspect	Pathophysiological Mechanisms	Diagnostic Approach	Disease-Specific Treatments	Symptomatic Management Approaches
**Fabry disease**				
Abdominal pain, diarrhea and constipation Less common: vomiting, nausea and early satiety	Autonomic nervous system dysregulation vasculopathy Inflammation Dysbiosis Maldigestion of oligosaccharides	Enzyme activity (male), lysoGb3 and gene analysis If endoscopy is performed: Gb3 deposition in intestinal epithelial cells Specific score (FABPRO-GI)	ERT Chaperone (migalastat)	Symptomatic therapy Low FODMAP diet Dietary supplements containing oral α-Gal A
**Gaucher disease**				
Protein-losing enteropathy (abdominal pain, bloating, diarrhea, hypoproteinemia, oedema, weight loss and malnutrition)	Gaucheromas of the mesenteric lymph nodes block mesenteric lymphatic drainage, resulting in increased lymphatic pressure and intestinal lymphangiectasia	Increased fecal alpha1-antitripsin Abdominal US, CT and MRI Endoscopy: intestinal lymphangiectasia Biopsy if malignancy is suspected	ERT (limited efficacy) SRT	Supportive care (albumin infusion, fluid and electrolyte replacement, high-protein diet with MCT lipid supplementation and parenteral nutrition) Budesonide
Epigastric discomfort and weight loss (case report) GI bleeding	Mucosal infiltration	Endoscopy	ERT SRT	Symptomatic therapy Surgical treatment
**Pompe disease**				
IOPD: feeding difficulties and failure to thrive	Smooth muscle involvement–dysmotility	Swallowing study (videofluoroscopic or fiberoptic endoscopic evaluation) Evaluation for GERD (pH-impedance monitoring)	ERT	Gastrostomy
LOPD: abdominal pain; feeding and swallowing difficulties; gastro-esophageal reflux; postprandial bloating; early satiety; abdominal discomfort; chronic diarrhea; constipation; poor weight gain; and decreased gag reflex	Smooth muscle involvement–dysmotility	Based on clinical manifestations	ERT	Symptomatic therapy Gastrostomy
LOPD: abdominal pain and colonic volvulus	Hepatodiaphragmatic interposition of the intestine	Abdominal Rx: Chilaiditi’s sign	ERT	Surgical treatment for intestinal obstruction
IOPD and LOPD: fecal urgency and incontinence	Striated and smooth pelvic floor muscle weakness Lower motor neuron involvement Autonomic involvement	Electromyography Rectal manometry	ERT	
**Niemann–Pick C**				
Inflammatory bowel disease	Reduced STING (stimulator of interferon genes) Impaired NOD2-mediated bacterial killing	Fecal calprotectin Endoscopy		Inducing autophagy to rescue bacterial handling Biologic therapy targeting cytokines
Diarrhea, nausea, bloating, abdominal pain and weight loss	Disaccharidase deficiency	Duodenal biopsy		Low-carbohydrate diet
Diarrhea, bloating and abdominal pain	Inhibition of intestinal α-glucosidases by miglustat	Lactose breath test		Low-carbohydrate diet
**Mucopolysaccharidoses**				
Diarrhea, dysphagia, constipation, loss of bowel control and fecal incontinence Less common: gastrointestinal bleeding, gastroenteritis, paralytic ileus and CIPO	Infiltration of the GI tract and Auerbach nerve plexuses by GAGs Dysbiosis	Based on clinical manifestations	ERT, if available	Symptomatic therapy Surgical treatment Gastrostomy
Chronic diarrhea	Obstruction of the intestinal lymphatics with intestinal lymphangiectasia	Endoscopy	ERT, if available	Low-fat diet supplemented with MCT
**Wolman disease**				
Malabsorption and diarrhea	Infiltration of the intestinal wall by foamy histiocytes	Abdominal US, CT and endoscopy	ERT	
Chron like (1 case report)	Infiltration of the intestinal wall by foamy histiocytes	Endoscopy	ERT	

## Data Availability

No new data were created or analyzed in this study.
